# Effects of sugammadex on heart rate variability in anesthetized male rats

**DOI:** 10.14814/phy2.70507

**Published:** 2025-08-14

**Authors:** Mika Nishikawa, Toru Kawada, Keita Saku, Hiroyuki Kinoshita, Shinji Kawahito

**Affiliations:** ^1^ Department of Dental Anesthesiology Tokushima University Hospital Tokushima Japan; ^2^ Department of Cardiovascular Dynamics National Cerebral and Cardiovascular Center Osaka Japan; ^3^ NTTR‐NCVC Bio Digital Twin Center National Cerebral and Cardiovascular Center Osaka Japan; ^4^ Department of Anesthesiology and Intensive Care Hamamatsu University School of Medicine Hamamatsu Japan

**Keywords:** arterial pressure, power spectra, rocuronium, R‐R interval, vagotomy

## Abstract

The administration of sugammadex has been associated with bradycardia and rarely asystole; however, the underlying mechanisms remain unclear. We examined heart rate variability (HRV) as an index of autonomic nervous system activity and investigated the effects of sugammadex on HRV in α‐chloralose and urethane anesthetized male rats. In protocol 1, we examined the effect of intravenous administration of sugammadex (10 mg/kg) alone. In protocol 2, sugammadex was administered 3 min after the administration of rocuronium bromide. In protocol 3, the effect of sugammadex was assessed after vagotomy. Sugammadex significantly prolonged the mean R‐R interval and increased time domain HRV indices, total power, high‐frequency power, and low‐frequency power. Rocuronium prior to sugammadex did not affect sugammadex‐induced percent changes in HRV indices. Prior vagotomy abolished the effects of sugammadex on HRV. These results suggest that sugammadex prolonged the mean R‐R interval and augmented HRV indices via vagal activation.

## 1 INTRODUCTION

Sugammadex is a modified gamma‐cyclodextrin that reverses steroidal non‐depolarizing neuromuscular blockers (NMBs), such as rocuronium and vecuronium, by encapsulating plasma free NMBs. Conventional reversal agents, including neostigmine, inhibit acetylcholinesterase and increase the concentration of acetylcholine at the neuromuscular junction to reverse the effects of NMBs; however, they are associated with muscarinic side effects, including bradycardia, hypersalivation, bronchoconstriction, nausea, and vomiting. In contrast, sugammadex exhibits very high affinity to rocuronium, forming the sugammadex‐rocuronium inclusion complex, which reduces the concentration of plasma free rocuronium and promotes its clearance from the neuromuscular junction. Consequently, sugammadex does not induce an excessive increase in the concentration of acetylcholine at the neuromuscular junction and is less likely to cause muscarinic side effects. During the development of sugammadex, animal studies did not report a significant effect of sugammadex on heart rate (HR) (Booij et al., 2009; de Boer et al., 2006). Nevertheless, there are multiple case reports of patients who developed severe bradycardia or cardiac arrest after the administration of sugammadex (Fierro et al., 2021; Hunter & Naguib, 2018; Pereira et al., 2023; Sims et al., 2020; Teng et al., 2021). The bradycardic side effect is not directly explained by the mode of action of sugammadex to encapsulate rocuronium. A recent study suggested the involvement of autonomic mechanisms in HR changes after the administration of sugammadex (Ebert et al., 2022).

In the present study, we assessed HR variability (HRV) as an index of autonomic nervous system activity before and after the administration of sugammadex in anesthetized rats. We tested the hypothesis that sugammadex caused bradycardia by shifting the autonomic balance toward vagal predominance in anesthetized rats.

## 2 METHODS

Experiments conformed to the “Guiding Principles for the Care and Use of Animals in the Field of Physiological Sciences”, which has been approved by the Physiological Society of Japan. All experimental protocols were reviewed and approved by the Animal Subjects Committee at the National Cerebral and Cardiovascular Center (#22033, #23013, and #24057).

### 2.1 Animal preparation

Male Sprague–Dawley rats were purchased from Japan SLC, Inc. (Shizuoka, Japan) and maintained on a 12‐h light–dark cycle with free access to water and standard laboratory chow (CLEA Japan, Inc., Tokyo, Japan). The rats [425 ± 40 g, 13.6 ± 1.0 weeks of age, mean ± standard deviation (SD)] were anesthetized with an intraperitoneal injection (2 mL/kg) of a mixture of α‐chloralose (40 mg/mL) and urethane (250 mg/mL), followed by the continuous intravenous infusion of a maintenance dose of the anesthetic drug mixture (18‐fold dilution with physiological saline, 2 mL·kg^−1^·h^−1^) through a polyethylene catheter (PE‐50, Becton Dickinson and Company, Sparks, MD, USA), which was inserted into the left femoral vein. Another catheter (PE‐50) was inserted into the right femoral vein for the administration of test drugs. An arterial catheter (PE‐50) was inserted into the right femoral artery to monitor arterial pressure (AP) using a fluid‐filled pressure transducer (DX‐200, Nihon Kohden, Tokyo, Japan). Ringer's lactate solution was continuously administered (4 mL·kg^−1^·h^−1^) to maintain fluid balance. Rats were intubated through tracheotomy and ventilated artificially at approximately 80 cycles/min and a tidal volume of approximately 2.4 mL with oxygen‐enriched air. Surface electrocardiography (ECG) was performed using a signal amplifier with a bandpass filter between 150 and 1000 Hz (AB‐610J, Nihon Kohden), and HR was monitored using a cardiotachometer (AT‐601G, Nihon Kohden). Body temperature was maintained at approximately 37°C using a heating pad and lamp.

### 2.2 Protocols

In protocol 1 (*n* = 8 rats), we examined the effects of the intravenous administration of sugammadex alone. Baseline data were acquired for more than 30 min after the surgical preparation had been completed. Sugammadex (CAS RN 343306‐79‐6, Tokyo Chemical Industry, Tokyo, Japan; dissolved with physiological saline to 10 mg/mL) was administered intravenously at 10 mg/kg as a bolus injection, and hemodynamic changes were monitored for approximately 30 min. The dose of sugammadex exceeded the typical clinical dose of 2–4 mg/kg in humans, which may have made the effects of sugammadex on HRV more easily detectable. Next, we performed bilateral vagotomy at the neck to remove the effects of vagal nerves on HRV and observed hemodynamic changes for 15–20 min. Finally, we administered the ganglionic blocker hexamethonium bromide (CAS RN 55‐97‐0, FUJIFILM Wako Pure Chemical Corporation, Osaka, Japan; dissolved with physiological saline to 30 mg/mL) at 60 mg/kg and monitored hemodynamic changes for approximately 30 min until HR reached intrinsic HR, which is HR in the absence of autonomic control. To estimate HRV under stable hemodynamics, a 5‐min data segment was selected during baseline and near the end of each condition (after sugammadex, vagotomy, and hexamethonium). In addition, the HRV analysis was performed at an earlier time point (5 min) after sugammadex administration, though the results may have been confounded by the volume loading effect.

In protocol 2 (*n* = 8 rats), we investigated whether the administration of rocuronium prior to sugammadex significantly affected hemodynamic responses. After baseline data were acquired for more than 30 min, rocuronium bromide (CAS RN 119302‐91‐9, FUJIFILM Wako Pure Chemical Corporation; dissolved with physiological saline to 5 mg/mL) was administered intravenously (5 mg/kg), which is 5 times the typical clinical dose in humans. Three min later, sugammadex was added intravenously (10 mg/kg). The effects of rocuronium plus sugammadex on hemodynamics were analyzed using a stable 5‐min segment near the end of a 30‐min observation period.

In protocol 3 (*n* = 7 rats), we investigated the effects of prior vagotomy on the hemodynamic responses to sugammadex. Bilateral vagotomy was performed following the introduction of the venous and arterial lines. After hemodynamic stabilization, baseline data were acquired. Thereafter, sugammadex was administered intravenously (10 mg/kg). The effects of sugammadex after vagotomy were analyzed using a stable 5‐min segment near the end of a 30‐min observation period.

At the end of the experiment, rats were euthanized by an intravenous injection of a saturated solution of potassium chloride under deepened anesthesia.

### 2.3 Data analysis

Data were sampled at 1000 Hz using a 16‐bit analog‐to‐digital converter [AD16‐16(PCI)EV, Contec, Osaka, Japan] and stored on a dedicated laboratory computer system. The R wave of ECG was detected offline using custom‐made software, and the R‐R interval sequence was analyzed. Time‐domain and frequency‐domain indices of HRV were calculated from a 5‐min data segment.

Outliers in R‐R intervals were removed using a Hampel identifier (Davis & Gather, 1993), which detects outliers based on the median absolute deviation. The mean R‐R interval and the following time‐domain indices of HRV were then calculated: the standard deviation (SDRR), coefficient of variation (CVRR), and root mean square of successive differences (RMSSD) in R‐R intervals. Mean AP was calculated for the same period as the R‐R interval analysis.

To calculate the frequency‐domain indices of HRV, the R‐R interval sequence was linearly interpolated at 100 Hz. The interpolated R‐R signal was then segmented into 12 half‐overlapping segments with a length of 2^12^ (4096) points each. In each segment, power spectra were calculated using fast Fourier transform after removing the linear trend and applying a Hanning window. Power spectra were ensemble averaged over the 12 segments to obtain total power (TP: 0.024–2.4 Hz), low‐frequency power (LF: 0.024–0.6 Hz), and high‐frequency power (HF: 0.6–2.4 Hz) (Kuo et al., 2004).

### 2.4 Statistical analysis

In protocol 1, the time‐domain and frequency‐domain parameters of HRV were compared across five time points: baseline, the early time point of sugammadex administration (starting 5 min after administration), the late time point of sugammadex administration (near the end of the 30‐min observation period), after vagotomy, and after hexamethonium administration. We examined the homogeneity of variance among the five time points using the Levene median test (Glantz & Slinker, 2001). When the dataset passed the equal variance test, we performed a repeated measures one‐way analysis of variance (ANOVA). The residuals of ANOVA were tested for normality using the D'Agostino‐Pearson test (D'Agostino et al., 1990). When the residuals passed the normality test, we accepted the results of ANOVA; otherwise, we changed the analysis to the nonparametric Friedman test. We also performed the nonparametric Friedman test when the dataset did not pass the initial test of the homogeneity of variance.

When there was an overall difference across the five conditions by the repeated measures one‐way ANOVA or Friedman test, a post hoc analysis was performed using a bootstrap test, which does not assume a normal distribution of tested statistical quantity (Efron & Tibshirani, 1994; Kawada et al., 2016, 2019). The bootstrap test for paired data was performed against the baseline condition, and the significance level was adjusted according to Holm's method (Glantz, 2012). Briefly, for a significance level *α*, the smallest *p* value was compared with α/4, the second smallest with α/3, the third smallest with α/2, and the largest with α. The comparison was terminated prematurely when the preceding comparison was not significant.

In protocol 2, the difference between the baseline and after the administration of rocuronium plus sugammadex was examined using the bootstrap test for paired data. Furthermore, percent changes in HRV parameters relative to baseline values were compared between protocols 1 and 2 using the bootstrap test for unpaired data.

In protocol 3, which was performed under vagotomized conditions, the difference between before and after the administration of sugammadex was examined using the bootstrap test for paired data. In all statistical analyses, differences were considered significant at *p* < 0.05.

## 3 RESULTS

**FIGURE 1:**
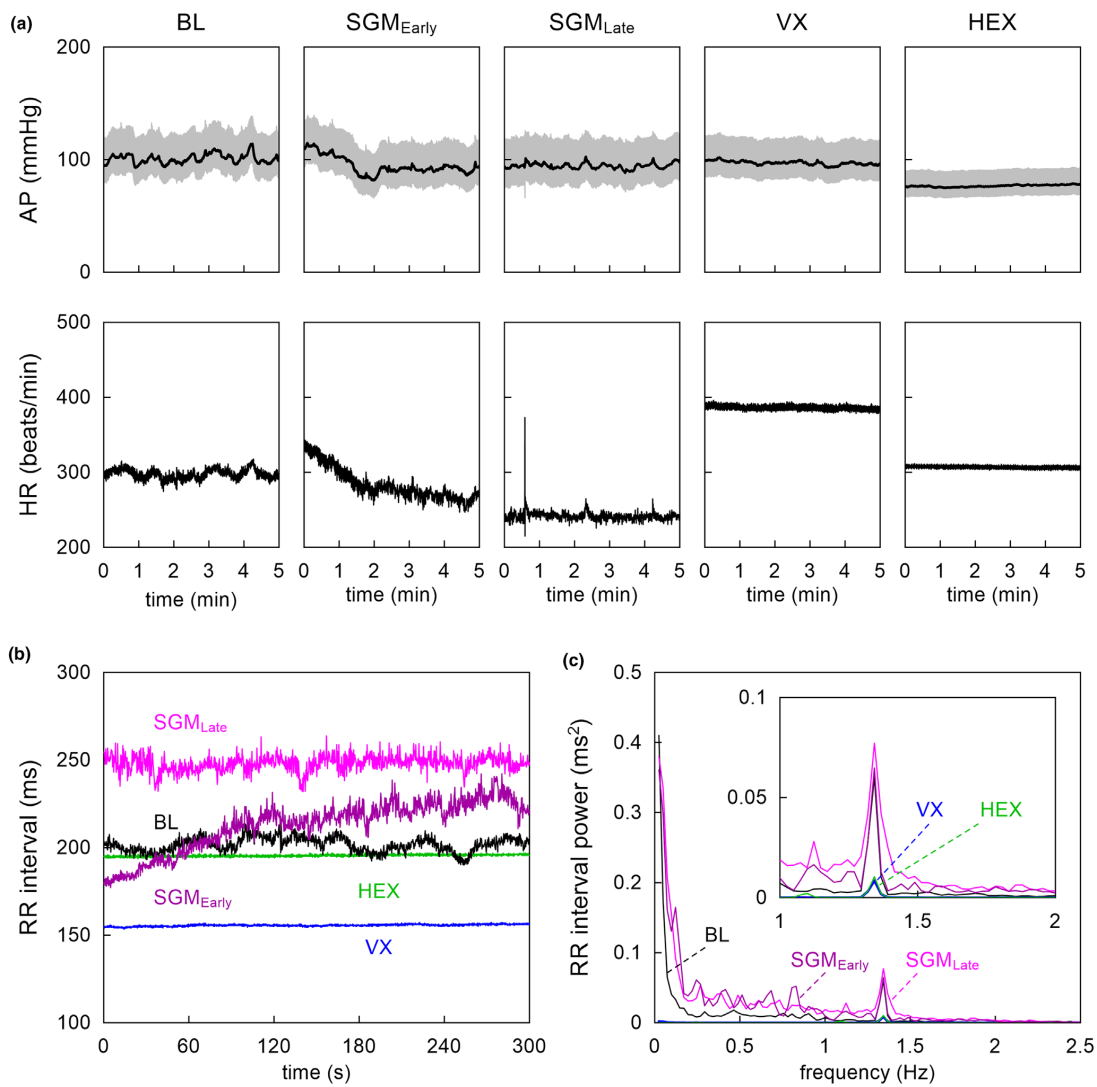
(a) Experimental data obtained from a rat in protocol 1. The arterial pressure (AP) is depicted as 100‐Hz resampled (gray) and 2‐s moving averaged (black) data. The heart rate (HR) is depicted as 10‐Hz resampled data. (b) The R‐R interval signal after removing outliers. (c) Power spectra of R‐R intervals. The inset shows an enlarged scale around the peak of power spectra. BL, baseline; HEX, hexamethonium; SGM_Early_, early time point after sugammadex; SGM_Late_, late time point after sugammadex; VX, vagotomy.

Figure 1a shows 5‐min traces obtained from a rat in protocol 1. At the early time point of sugammadex administration, HR showed a decreasing trend. Compared to baseline, sugammadex reduced the mean HR at the late time point but did not significantly affect the mean AP. Vagotomy increased the mean HR without significantly affecting the mean AP. Hexamethonium decreased the mean AP and reduced the mean HR back towards the baseline level. Figure 1b depicts R‐R interval signals after the removal of outliers. The R‐R interval signal under the baseline condition showed some HRV. Sugammadex prolonged the mean R‐R interval and increased HRV. Vagotomy shortened the mean R‐R interval and nearly abolished HRV. Hexamethonium prolonged the mean R‐R interval back towards the baseline value, without restoring HRV. Figure 1c shows the power spectra of R‐R intervals. Under the baseline condition (black lines), the power of the LF range increased from below 0.25 Hz towards 0 Hz. The power of the HF range showed a peak at approximately 1.3 Hz. Sugammadex increased the power spectra over the entire frequency range (dark purple and magenta lines). Vagotomy significantly reduced power over the entire frequency range (blue lines), resulting in nearly no power, except for a peak at approximately 1.3 Hz. Hexamethonium did not induce further changes in the power spectra in this animal (green lines).

**FIGURE 2:**
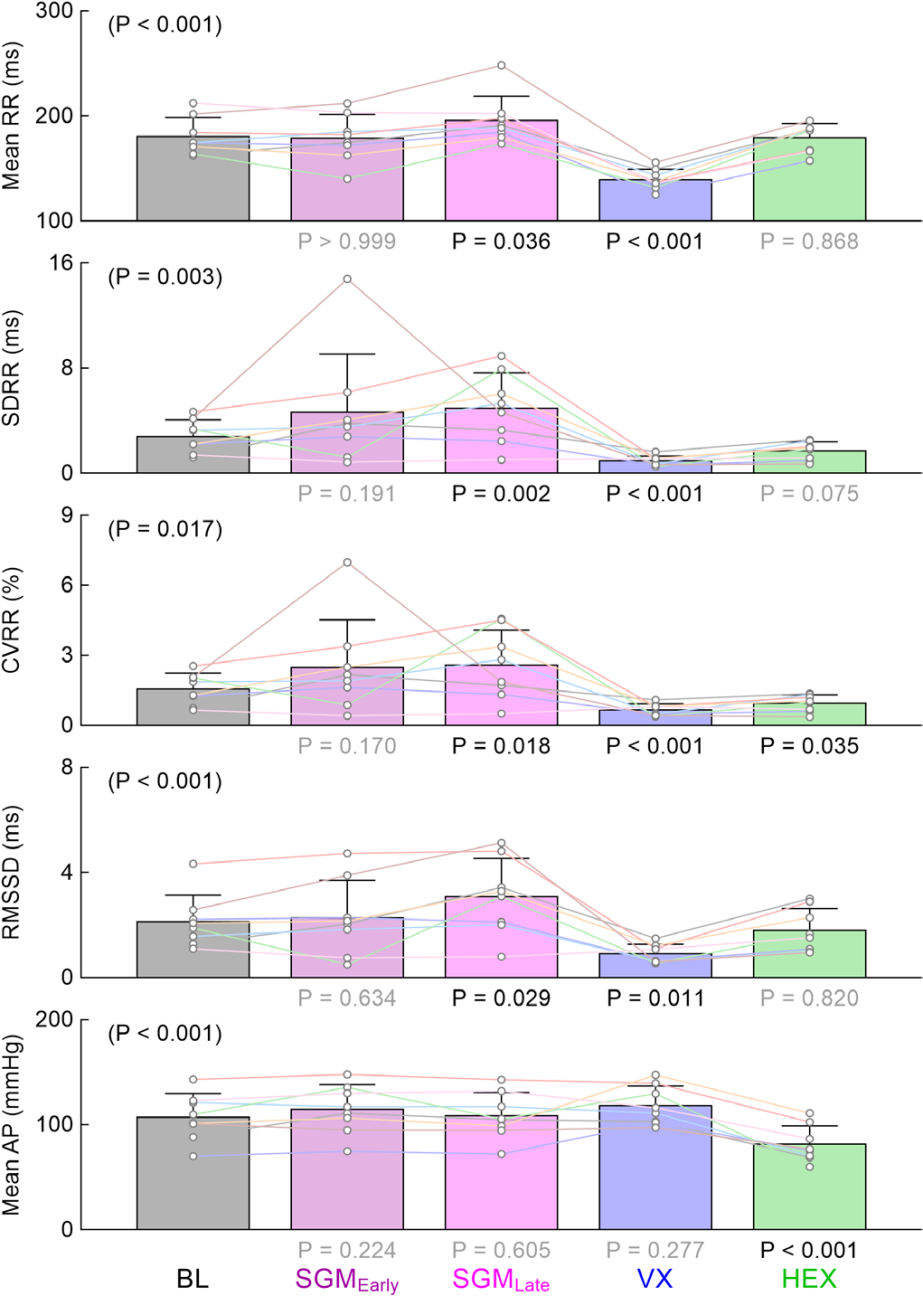
The mean R‐R interval, time‐domain indices of heart rate variability [standard deviation (SDRR), coefficient of variation (CVRR), and root mean square of successive differences (RMSSD) in R‐R intervals], and mean arterial pressure (AP) obtained at baseline (BL), early time point after the administration of sugammadex (SGM_Early_), late time point after the administration of sugammadex (SGM_Late_), after vagotomy (VX), and after the administration of hexamethonium (HEX) in protocol 1. The *p* value in the parenthesis was derived from a repeated measures analysis of variance or the Friedman test among the five time points examined, depending on the homogeneity of variance. Bar graphs show the mean ± SD values with data points for the respective rats (*n* = 8). The *p* values under the bar graphs were determined by the bootstrap test with Holm's correction for 4 simultaneous comparisons relative to BL. Statistically significant values (*p* < 0.05) are shown in black, while nonsignificant values are shown in gray.

Figure 2 summarizes changes in the mean R‐R interval, the time‐domain indices of HRV, and mean AP among the five time points obtained in protocol 1. Although mean values of SDRR and CVRR at the early time point of sugammadex administration showed changes in the same direction as those at the late time point, these changes were not statistically significant compared to baseline. Sugammadex significantly prolonged the mean R‐R interval and increased SDRR, CVRR, and RMSSD at the late time point. Vagotomy significantly shortened the mean R‐R interval and decreased SDRR, CVRR, and RMSSD. While the mean R‐R interval, SDRR, and RMSSD after the administration of hexamethonium were not significantly different from the respective baseline values, CVRR after hexamethonium remained lower than that in the baseline condition. Sugammadex and vagotomy did not significantly affect mean AP from that under the baseline condition, whereas hexamethonium significantly decreased mean AP.

**FIGURE 3:**
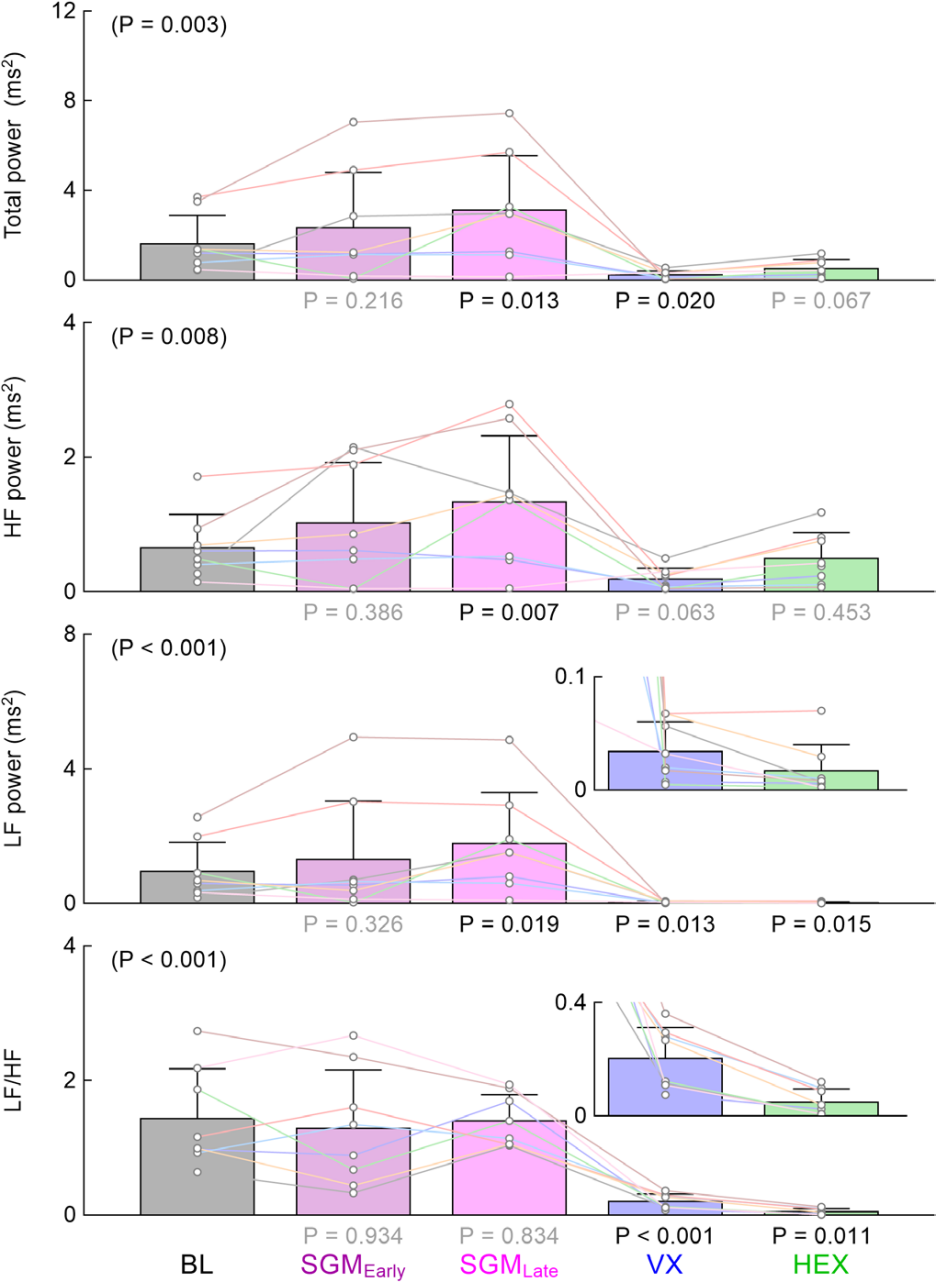
Frequency‐domain indices of heart rate variability. Total power, high‐frequency (HF) power, low‐frequency (LF) power, and LH/HF obtained at baseline (BL), early time point after the administration of sugammadex (SGM_Early_), late time point after the administration of sugammadex (SGM_Late_), after vagotomy (VX), and after the administration of hexamethonium (HEX) in protocol 1. The *p* value in the parenthesis was derived from a repeated measures analysis of variance or the Friedman test among the five time points, depending on the homogeneity of variance. Bar graphs show the mean ± SD values with data points for the respective rats (*n* = 8). The *p* values under the bar graphs were determined by the bootstrap test with Holm's correction for 4 simultaneous comparisons relative to BL. Statistically significant values (*p* < 0.05) are shown in black, while nonsignificant values are shown in gray.

Figure 3 shows the frequency‐domain indices of HRV among the five time points obtained in protocol 1. Although mean values of TP, HF power, and LF power at the early time point of sugammadex administration showed changes in the same direction as those at the late time point, these changes were not statistically significant compared to baseline. Sugammadex significantly increased TP, HF power, and LF power at the late time point, but did not significantly affect LF/HF. Vagotomy significantly reduced TP, LF power, and LF/HF. TP, LF power, and LF/HF remained lower after the administration of hexamethonium than the respective baseline values. HF power after the administration of hexamethonium was not significantly different from the baseline value.

**FIGURE 4:**
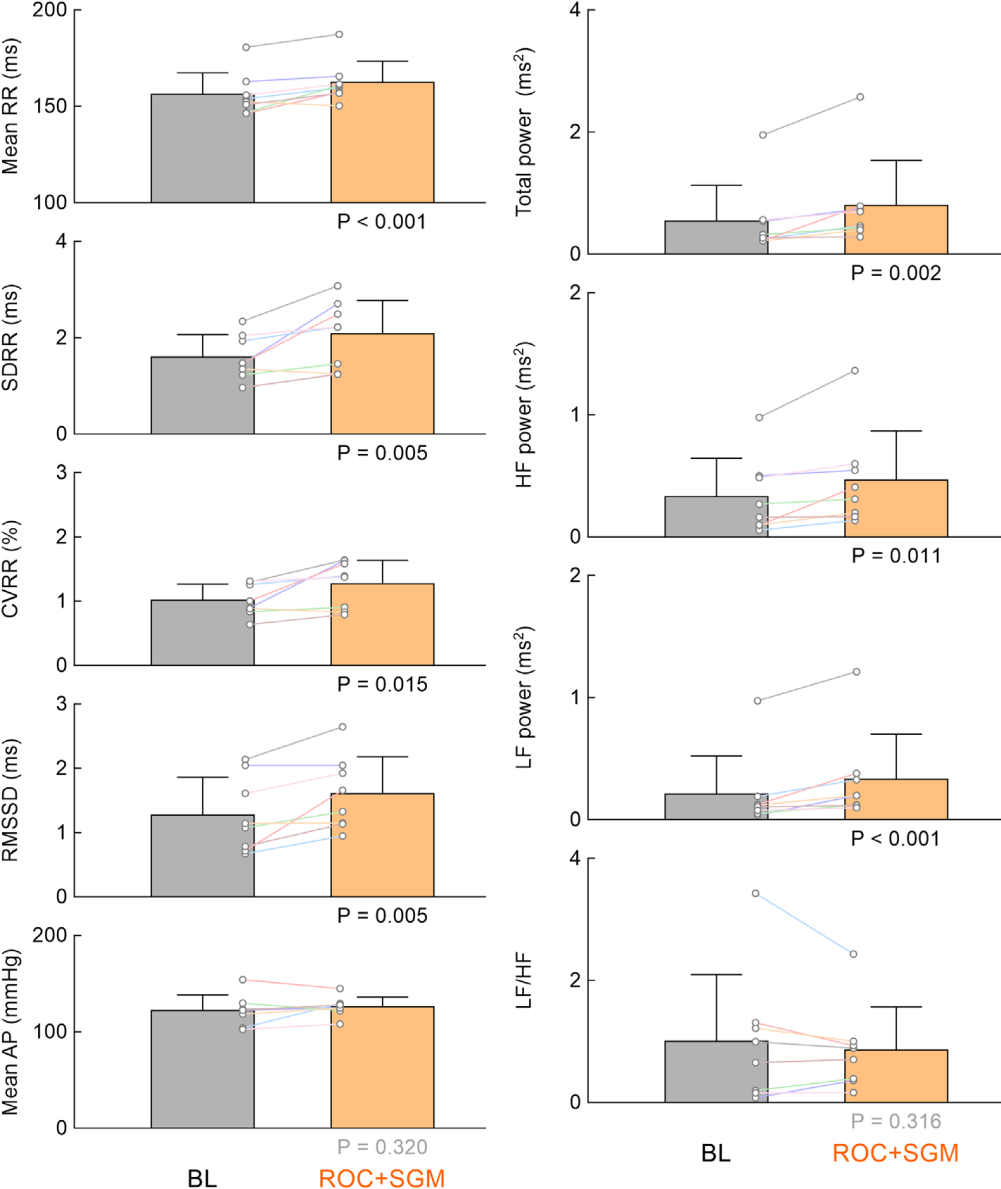
The mean R‐R interval, time‐domain indices of heart rate variability [standard deviation (SDRR), coefficient of variation (CVRR), and root mean square of successive differences (RMSSD) in R‐R intervals], and mean arterial pressure (AP), as well as total power, high‐frequency (HF) power, low‐frequency (LF) power, and LF/HF obtained at baseline (BL) and after the administration of rocuronium plus sugammadex (ROC+SGM) in protocol 2. Bar graphs show the mean ± SD values with data points for the respective rats (*n* = 8). The *p* values under the bar graphs were determined by the bootstrap test for paired data. Statistically significant values (*p* < 0.05) are shown in black, while nonsignificant values are shown in gray.

Figure 4 summarizes the results of protocol 2. Rocuronium plus sugammadex significantly increased the mean R‐R interval without significantly affecting mean AP. Rocuronium plus sugammadex significantly increased the time‐domain indices of HRV, TP, HF power, and LF power, but did not significantly affect LF/HF.

Table 1 summarizes sugammadex‐induced percent changes in HRV parameters from their baseline values in protocols 1 and 2. The absolute baseline values, except for the HF power and LF/HF, were significantly smaller in protocol 2 (rocuronium plus sugammadex) than in protocol 1 (sugammadex alone). Although the mean values for percent changes were numerically smaller in protocol 2 than in protocol 1, except for LF/HF, none of the differences were significant; indicating that differences were within interindividual variations.

**TABLE 1:** Percent changes in heart rate variability indices relative to the baseline values in protocols 1 and 2.

	Protocol 1 (*n* = 8) SGM	Protocol 2 (*n* = 8) ROC+SGM	*p* Value
Baseline R‐R interval, ms	180.4 ± 17.9	156.2 ± 11.2	<0.001
Mean R‐R interval change	8.6 ± 8.4%	4.0 ± 3.3%	0.138
Baseline SDRR, ms	2.80 ± 1.26	1.60 ± 0.46	0.006
SDRR change	79.5 ± 77.9%	31.2 ± 31.1%	0.082
Baseline CVRR	1.55 ± 0.68%	1.02 ± 0.25%	0.021
CVRR change	64.9 ± 70.3%	26.0 ± 29.7%	0.126
Baseline RMSSD, ms	2.13 ± 1.01	1.27 ± 0.59	0.039
RMSSD change	49.6 ± 62.9%	35.6 ± 42.0%	0.570
Baseline total power, ms^2^	1.61 ± 1.29	0.54 ± 0.59	0.029
Total power change	122.8 ± 197.5%	70.2 ± 80.9%	0.438
Baseline HF power, ms^2^	0.66 ± 0.49	0.33 ± 0.31	0.105
HF power change	115.0 ± 161.0%	82.6 ± 110.6%	0.605
Baseline LF power, ms^2^	0.95 ± 0.86	0.21 ± 0.31	0.023
LF power change	148.2 ± 266.6%	118.3 ± 134.5%	0.715
Baseline LF/HF	1.43 ± 0.74	1.00 ± 1.09	0.320
LF/HF change	10.9 ± 39.0%	49.1 ± 135.4%	0.410

*Note*: The data are mean ± SD values. The *p* values were derived from the bootstrap test for unpaired data.

Abbreviations: CVRR, coefficient of variation of the R‐R intervals; HF power, high‐frequency power; LF power, low‐frequency power; LF/HF, the ratio of LF to HF; RMSSD, root mean squares of successive differences of the R‐R intervals; ROC, rocuronium; SDRR, standard deviation of the R‐R intervals; SGM, sugammadex (late time point).

**FIGURE 5:**
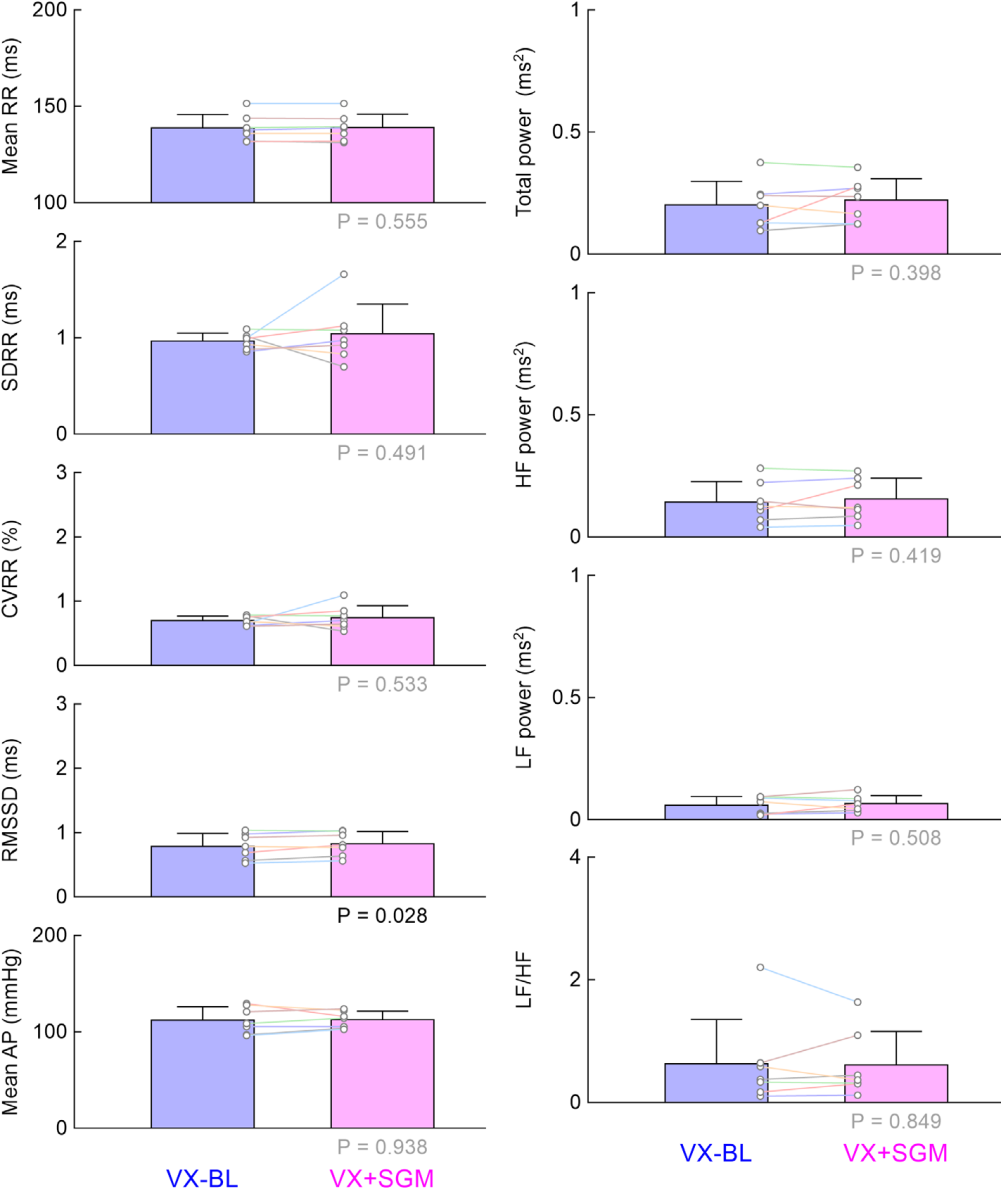
The mean R‐R interval, time‐domain indices of heart rate variability [standard deviation (SDRR), coefficient of variation (CVRR), and root mean square of successive differences (RMSSD) in R‐R intervals], and mean arterial pressure (AP), as well as total power, high‐frequency (HF) power, low‐frequency (LF) power, and LF/HF obtained at baseline (VX‐BL) and after the administration of sugammadex (VX + SGM) under vagotomized conditions in protocol 3. Bar graphs show the mean ± SD values with data points for the respective rats (*n* = 7). The *p* values under the bar graphs were determined by the bootstrap test for paired data. Statistically significant values (*p* < 0.05) are shown in black, while nonsignificant values are shown in gray.

Figure 5 summarizes the results of protocol 3. Prior vagotomy abolished the effects of sugammadex, except for the effect on RMSSD. Despite the statistical significance, the magnitude of the change in RMSSD was small (0.041 ± 0.048 ms, mean ± SD) and may have little pharmacological meaning.

## 4 DISCUSSION

The results obtained suggest that sugammadex induced significant bradycardia with increased indices of HRV. Rocuronium administration prior to sugammadex did not significantly modify the effects of sugammadex on HRV indices. Prior vagotomy abolished the effects of sugammadex on HRV indices.

### 4.1 Effects of sugammadex on HRV

In the present study, sugammadex significantly decreased mean HR and increased HRV indices in protocols 1 and 2. The increase in HF power after sugammadex was prevented by prior vagotomy in protocol 3. These results suggest that sugammadex increased the vagal nerve activity to the heart. On the other hand, the lack of significant changes in LF/HF and mean AP indicate that sugammadex did not significantly enhance sympathetic outflow.

The HR slowing suggests the activation of the efferent vagal nerve via the central and/or peripheral effect of sugammadex on vagal control mechanisms. The direct central effect of sugammadex seems incompatible with its very low movement across the blood–brain barrier (Palanca et al., 2013). Another explanation for vagal activation is that the rapid dissociation of rocuronium from nicotinic receptors after the administration of sugammadex evoked afferent signaling to affect autonomic tone (Ebert et al., 2022). However, percent changes in HR and HRV indices after the administration of sugammadex in combination with rocuronium in protocol 2 (Figure 4) were not significantly different from those in protocol 1 (Table 1). Therefore, the dissociation of rocuronium from nicotinic receptors at the neuromuscular junction may not explain bradycardia following the administration of sugammadex in the present study.

### 4.2 Effects of vagotomy and autonomic blockade on HRV

To confirm that bradycardia observed after the administration of sugammadex was dependent on intact vagal nerves, bilateral vagotomy was performed. As expected, vagotomy significantly decreased the mean R‐R interval and time‐domain and frequency‐domain indices of HRV (Figures 2 and 3). Although these results suggest that the vagal control of the heart was maintained under α‐chloralose and urethane anesthesia, plasma levels of sugammadex might have fallen substantially at the time of vagotomy in protocol 1. Nevertheless, because prior vagotomy in protocol 3 also abolished the effect of sugammadex on HRV, it may be reasonable to interpret that bradycardia observed after sugammadex depended on intact vagal nerves.

The HF components of HRV oscillate in synchrony with the respiratory rate. In conscious Wistar rats, the peak of the HF component was associated with spontaneous respiration (respiratory sinus arrythmia, RSA) at approximately 1.855 Hz (Japundzic et al., 1990). In the present study, RSA was observed at approximately 1.33 Hz (Figure 1c, black), which corresponded to the rate of artificial ventilation (80 cycles/min). Although the artificial ventilation rate may have been different from the native respiratory rate, the respiratory system entrains to mechanical ventilation via the Hering–Breuer reflex (Petrillo et al., 1983). Bilateral vagotomy significantly decreased RSA, but did not completely abolish RSA (Figure 1c, blue). In volume‐controlled, positive pressure ventilation in the present study, a mechano‐sensitive mechanism may have affected HR in response to changes in the central venous pressure fluctuating at the artificial ventilation rate (Kohl et al., 1999).

In the present study, vagotomy significantly reduced TP, HF power, and LF power. Since vagal nerves convey dynamic HR control in the frequency range of between 0.01 and 1 Hz in rats (Kawada et al., 2019), it is reasonable to assume that vagotomy decreased not only HF power but also LF power. In conscious rats, muscarinic blockade by atropine also decreased both HF and LF powers (Japundzic et al., 1990). However, the interpretation of LF/HF is complicated. While LF/HF has been suggested to reflect sympatho‐vagal balance in humans and conscious dogs (Pagani et al., 1986), LF/HF significantly decreased after vagotomy under the present experimental conditions, despite the removal of vagal modulation. Dynamic sympathetic modulation under anesthesia may have been very small both before and after vagotomy, making a proper assessment of the sympatho‐vagal balance unfeasible. However, this does not mean the absence of tonic sympathetic nerve activity after vagotomy and before the administration of hexamethonium in protocol 1 because hexamethonium significantly decreased mean AP. The administration of hexamethonium also prolonged the mean R‐R interval towards the baseline value, indicating that the reduced mean R‐R interval after vagotomy was partly due to the tonic sympathetic effect. These results suggest that the interpretation of LF/HF is difficult under anesthetized conditions.

## 5 LIMITATIONS

There are a number of limitations that need to be addressed. First, the study was not placebo‐controlled, which hampers drawing definitive conclusions regarding the effects of sugammadex on HRV. Second, since the present study examined rats, the results obtained may not be directly extrapolated to the reversal of NMBs in humans. Nevertheless, the rats may be used to examine the vagal effect on the heart because strong electrical vagal nerve stimulation can cause cardiac arrest as an extension of severe bradycardia (Kawada et al., 2020). Third, due to the small animal size, the administration of a test drug at 1 mL/kg induced acute variations in AP and HR via the volume loading effect. To avoid the phase of acute hemodynamic variations, we evaluated the effects of sugammadex 25–30 min after its administration. Although the evaluation period was within the half‐life of sugammadex (approximately 30 min) estimated in Rhesus monkeys using a dose of 1 mg/kg (de Boer et al., 2006), plasma drug concentrations should have been measured. Moreover, the dose of sugammadex was selected based on a previous study that examined the dose–response curve (Haerter et al., 2015), which indicated that 10 mg/kg sugammadex effectively reversed the effects of intravenous rocuronium at 3.5 mg/kg in rats. Effects on HRV may differ depending on the dose of sugammadex and/or rocuronium and species. Fourth, we did not perform a protocol to examine the effect of rocuronium alone. Although the results of protocol 2 suggest that rocuronium did not significantly modify the effect of sugammadex on HRV, adding a protocol with rocuronium alone might have strengthened the study. Finally, anesthetics used in the present study (α‐chloralose and urethane) differed from volatile agents commonly used in humans, such as sevoflurane. It is possible that the severe bradycardia observed during the reversal of rocuronium with sugammadex is related to the use of volatile anesthetics. Further studies are warranted to address these unresolved questions.

## 6 CONCLUSION

Sugammadex significantly prolonged the mean R‐R interval and augmented indices of HRV from baseline conditions, suggesting vagal activation. The administration of sugammadex in combination with rocuronium did not enhance the HR effect of sugammadex. Severe bradycardia described in case reports was not reproduced in anesthetized normal rats under the present experimental settings.

## AUTHOR CONTRIBUTIONS

MN and SK conceived the study. MN, TK, and SK designed the study. MN and TK performed the experiment and analyzed the data. MN, TK, KS, HK, and SK interpreted the data. MN and TK drafted the manuscript. MN, TK, KS, HK, and SK edited the manuscript and approved the final version of the manuscript.

## FUNDING INFORMATION

The present study was partly supported by the research program of the Japan Agency for Medical Research and Development (23hk0102085h0002), the research program of the Ministry of Internal Affairs and Communications (SCOPE: JP225006004), the Intramural Research Fund for Cardiovascular Diseases of National Cerebral and Cardiovascular Center (21‐2‐7, 21‐2‐9), a research grant from JST (JPMJPF2018), and a research grant from NTT Research, Inc. The authors confirm that these parties had no influence on the study design, contents of the article, or selection of this journal.

## CONFLICT OF INTEREST STATEMENT

Toru Kawada received a consulting fee from NTT Research, Inc. Keita Saku received research funding from Abiomed Japan K.K., NTT Research, Inc., Asahi Kasei ZOLL Medical Corporation, Neuroceuticals Inc., and Zeon Medical Inc., and honoraria from Abiomed Japan K.K. and Mallinckrodt Pharmaceuticals Inc. Other authors declare no conflicts of interest.

## ETHICS STATEMENT

Male Sprague–Dawley rats were purchased from Japan SLC, Inc. (Shizuoka, Japan). Experiments conformed to the “Guiding Principles for the Care and Use of Animals in the Field of Physiological Sciences”, which has been approved by the Physiological Society of Japan. All experimental protocols were reviewed and approved by the Animal Subjects Committee at the National Cerebral and Cardiovascular Center (#22033, #23013, and #24057).

## Data Availability

The datasets obtained in the present experiment are available from the corresponding author upon reasonable request.
